# Automated TEM Reveals Intercrystalline Correlations of Conjugated Polymers

**DOI:** 10.1021/acs.macromol.5c02888

**Published:** 2026-02-09

**Authors:** Ryan A. Fair, Dhruv Gamdha, Joshua T. Del Mundo, Abigail M. Fenton, Agatha O’Connell, Karen C. Bustillo, Esther W. Gomez, Andrew M. Minor, Baskar Ganapathysubramanian, Enrique D. Gomez

**Affiliations:** † Department of Materials Science and Engineering, 8082 The Pennsylvania State University, University Park, Pennsylvania 16802, United States; ‡ Department of Mechanical Engineering, 1177 Iowa State University, Ames, Iowa 50011, United States; § Department of Chemical Engineering, The Pennsylvania State University, University Park, Pennsylvania 16802, United States; ∥ Department of Biomedical Engineering, The Pennsylvania State University, University Park, Pennsylvania 16802, United States; ⊥ National Center for Electron Microscopy, Molecular Foundry, 1666 Lawrence Berkeley National Laboratory, Berkeley, California 94720, United States; # Department of Materials Science and Engineering, University of California, Berkeley, California 16802, United States; ¶ Translational AI Research Center (TrAC), Iowa State University, Ames, Iowa 50011, United States

## Abstract

Transmission electron microscopy (TEM) continues to transform polymer science by revealing key aspects of chain packing, phase separation and nanoscale structure. The development of instrumentation and data analyses tools is driving the field forward and enabling new experiments. Here, we use automated high-resolution TEM (HRTEM) and image processing to identify the structure of a conjugated polymer used in organic electronics. Analysis of more than 600 HRTEM images reveals lattice parameters and orientation correlations between crystals, including the preferred alignment of neighboring crystals along the same crystallographic direction that is likely the result of liquid crystalline order.

## Graphical abstract

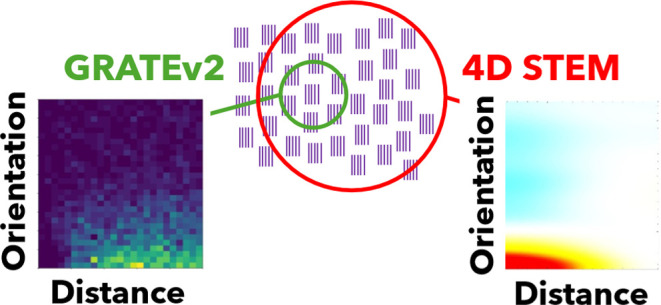

Transmission electron microscopy (TEM) is a powerful tool for characterizing chain packing and nanoscale structure in polymers.^1–3^ Imaging using TEM led to insights on the factors that govern adhesion,^4^ yield strength,^5^ thermal response,^6^ phase behavior,^7^ permeability,^8^ and electrical conductivity;^9^ microscopy can also connect nanostructure and functionality of conjugated polymers in various organic electronic devices.^10–12^ In addition to qualitative observations, quantification of crystal domains,^11,13–16^ phase transitions,^17,18^ degree of crystallinity,^11,13^ particle size and distribution,^19,20^ mixing behavior,^15,18,21^ and dynamic response^22^ in polymer systems is possible, although previous work has relied on a limited number of TEM images.

Recent developments in TEM instrumentation, such as autosampling and computationally automated image processing,^14,23,24^ are revolutionizing the field of electron microscopy. Automated acquisition and single-particle reconstructions are resolving the structure of proteins and viruses with remarkable resolution, down to nearly an angstrom.^25^ The uniformity of proteins enables the combination of multiple data sets, sometimes from thousands of images, to computationally reconstruct the 3D structure at high resolution. Although the variability in the structure of polymers would seem to preclude this approach, recent work has demonstrated the application to polypeptoids with well-defined monomer sequence and minimal dispersity.^26^ Thus, a new approach for microscopy of disordered soft matter is emerging, which is enabled by multi-image acquisition and analyses.

Here, we combine automated TEM with an automated crystal feature extraction framework called Graph based Analysis of Transmission Electron Microscopy (GRATEv2)^24,27^ to reveal orientational correlations between crystals within a film of poly­[*N*-9′-heptadecanyl-2,7-carbazole-*alt*-5,5-(4′,7′-di-2-thienyl-2′,1′,3′-benzothiadiazole)] (PCDTBT, Figure 1). Previous work speculated that PCDTBT is amorphous,^28,29^ crystalline,^30^ or locally ordered.^31^ We show that the high glass transition temperature (*T*_g_) of PCDTBT requires elevated temperature annealing for order formation, and the combination of 4-dimensional scanning transmission electron microscopy (4D-STEM)^3,13^ and grazing incidence wide-angle X-ray scattering (GIWAXS) reveal various aspects of crystal structure and texturing within thin films. Analysis of over 600 images using a graph based analysis of TEM images (GRATEv2) identifies a preferential alignment of adjacent grains with the same crystallographic orientation. We hypothesize that liquid crystalline order in PCDTBT, which is evident from rheology, leads to orientational correlations that are apparent over hundreds of nanometers.

**Figure 1:**
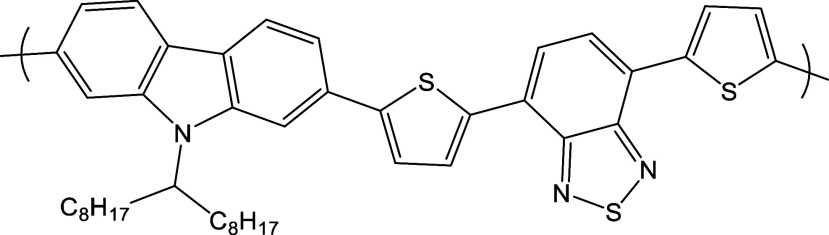
Chemical structure of PCDTBT.

## Materials and Methods

All materials were sourced from Sigma-Aldrich unless otherwise noted. PCDTBT was synthesized with a Suzuki polycondensation reaction using established methods.^32^ Molecular weight was characterized using an Agilent 1260 Infinity gel permeation chromatography system and previously reported methodologies for universal calibration-based measurements.^33^

To prepare TEM and GIWAXS samples, 5 mg/mL solutions of PCDTBT were dissolved in chlorobenzene inside a nitrogen glovebox at 45 °C for a minimum of 12 h. Silicon wafers were cleaned using sonication for 20 min in acetone and then 20 min in isopropanol. This was followed by UV-ozonation for 20 min. Poly­(3,4-ethylenedioxythiophene)-poly­(styrenesulfonate) (PEDOT/PSS) (Clevois P, H.C. Starck) films were cast on silicon substrates by spin-casting for 2 min at 4000 rpm in air, which serves as a sacrificial layer for film floating. Substrates were then brought into the nitrogen glovebox and PCDTBT films spun cast for 2 min at 800 rpm. Based on ellipsometry (J.A.Woollam RC-2 spectroscopic ellipsometer), the thickness of PCDTBT films was 32.3 ± 1.4 nm.

For TEM sample preparation, coated substrates were brought out of the glovebox and floated off in deionized water and subsequently collected onto copper TEM grids. Samples were left under ambient conditions to dry overnight, and then annealed in a nitrogen glovebox at 190 °C for 2 h. All HRTEM and 4D-STEM samples were prepared at the same time in bulk.

For GIWAXS sample preparation, PEDOT/PSS films deposited on silicon substrates was annealed at 165 °C for 10 min and was then coated with PCDTBT using the spin coating conditions described above. After PCDTBT coating, samples were left to dry overnight before being annealed in a nitrogen glovebox for 2 h.

HRTEM imaging was done at the Penn State Materials Characterization Lab on the FEI Titan Krios microscope operating at 300 kV using the K2 direct electron detector and a cryo-stage. Dose rate was adjusted to 20 e/Å^2^s for a 2.5 s exposure. Automated acquisition was set to autofocus before each acquisition at 300 kx magnification with a random defocus value ranging between 0 and −3 μm. Images were acquired at 470 kx magnification with a step size of 2.5 μm between exposed regions. HRTEM data used for GRATEv2 analysis was acquired across multiple samples to ensure sufficient data volume.

A total of 637 images were collected for GRATEv2 analysis. Analysis was done using the Python framework developed in Gamdha et al.^24,27^ Crystal segmentation within an image is achieved through a two-step process. First, the skeletonized morphological features of the crystal structure are extracted to capture their essential shapes and orientations. Next, a graph is constructed where each node represents a skeleton segment, and edges connect nodes with similar orientations and close spatial proximity. These graph-connected components are then filtered based on their node counts, enabling the precise delineation and segmentation of distinct crystalline regions within the image. Processing parameters were set according to Table 1. Analysis was performed on domains containing spacings from 1.7 to 2.4 nm, and 0.42 to 0.5 nm, which was used to characterize PCDTBT (100) spacings and (010) spacings, respectively. From this analysis, the following parameters were identified for each crystal: position coordinates of crystal center of mass, angle orientation relative to image axis, *d*-spacing, and crystal length along major and minor axis. From these parameters, we could calculate crystal area and intercrystallite relationships, such as separation distance and relative orientation. A total of 4350 ordered domains were identified from the HRTEM images using GRATEv2. A table of all these parameters for each identified crystal is available in the Supporting Information as an .xlsx file. Processing was executed on a computer equipped with a 96-core AMD EPYC 9654 CPU @ 3.7 GHz running on Linux OS. This completed analysis of the entire data set on the order of minutes.

**Table 1:** Input Parameters for GRATEv2 Algorithm

parameter	value
image resolution	78.5 (pixels/nm)
blurring iterations	15 (unitless)
Kernel size blurring	0.15 (fraction of *d*-spacing)
Kernel size closing	15 (unitless)
Kernel size opening	17 (unitless)
threshold pixel length	0.625 (fraction of *d*-spacing)
uniform breaking length	1.5 (*d*-spacing)
threshold ellipse aspect ratio	5 (unitless)
adjacency distance	2 (*d*-spacing)
adjacency angle	10 (°)
threshold cluster size	7 (unitless)
power spectrum threshold	1.15 (unitless)

To assess whether the area-weighted *d*-spacing distribution produced by GRATEv2 contained more than one crystalline population, *d*-spacings (Δ*d* = 0.03 nm) were analyzed using a one-dimensional Gaussian mixture model (GMM). Each bin center was treated as an observation and weighted by the total crystalline area assigned to that bin; weighted bins were expanded into repeated samples to enable mixture fitting in scikit-learn. Models containing 1–3 Gaussian components were fit by maximum likelihood, and the optimal model was selected using the Akaike Information Criterion (AIC) and Bayesian Information Criterion (BIC). A two-component GMM provided the best fit, identifying peaks at 1.99 and 2.16 nm. Measurement uncertainty of 10% was incorporated by estimating a global error variance and subtracting it from the observed component variances, confirming that peak separation—not peak width—drives the bimodal structure.

Separation distance between identified domains were compared with a random distribution of separation distances for images with a resolution of 4096 × 4096. This random distribution was created using a random number generator to assign pixel values between 0 and 4095 for *x* and *y* coordinates. This was done for two points, and then the separation distance was calculated. This process was repeated for 10^6^ iterations to ensure a completely random distribution. Then, the histogram of these values was scaled down such that the total count of domains would equal the amount counted in the measured data. The contrast between the two distributions was used to determine that the measured distribution was not random noise.

Comparing the relative angle between different crystal planes of domains identified in our images requires calculation of the separation distance to identify the possibility of overlapping domains. For areas in our images where two lattice spacings are apparent, we identify the centroid of a domain defined by the presence of a 0.46 or 2.0 nm lattice spacing (±20%). Instead of the direct centroid-to-centroid distance, we calculate an edge-to-edge distance defined as *r*_edge_ = *r*_centroid_-(*r*_1_ + *r*_2_) where *r*_centroid_ is the Euclidean distance between the centroids of two crystals, and *r*_1_ and *r*_2_ are the effective radii. As such, *r*_edge_ < 0 indicates overlapping domains that are not perfectively aligned, *r*_edge_ > 0 indicates two crystals separated by an amorphous region, and *r*_edge_ = 0 indicates two crystals centered on top of each other or that the crystal planes originate from one crystal (see Figure S3 for demonstration). The relative angle, θ_r_, is simply the absolute difference between orientation angles, θ, of two identified ordered domains, as θ_r_ = |θ_1_ – θ_2_|. We use a kernel density estimator (KDE), which is a nonparametric statistical method, to estimate the probability density function of occurrences in which (010) and (100) spacings overlap. This was calculated using

1 $$\mathrm{f̂}\left(x\right)=\frac{1}{nh}\sum_{i=1}^{n}K\left(\frac{x-x_{i}}{h}\right)$$

For a set of observed instances (*x*_*i*_) in which *h* is the bandwidth smoothing parameter, *n* is the number of observations, and *K* is a Gaussian kernel function. In two dimensions, KDE was extended to jointly estimate the density over both *r* and θ_r_. At greater *r*, there is a linear increase in the likelihood of observed occurrences due to increase in observed area. To correct for geometric sampling bias, in which greater edge-to-edge distances inherently sample larger areas, we normalized each bin by its wedge area using binning widths determined by the Freedman–Diaconis rule.

4D-STEM data set collection was done using electron nanodiffraction on the TitanX microscope at the National Center for Electron Microscopy at Lawrence Berkeley National Laboratory. The microscope was operated at 300 kV with 20 and 70 μm C2 and C3 apertures, respectively. Step size between images was 10 nm, representing the lower resolution limit when characterizing long-range order. The diffusive effects of beam damage were limited using an exposure time of 0.197 s and a cryo-holder.^2^ Reported 4D-STEM data came from acquisition of a single film. Analysis was performed using MatLab based on previously established methods.^3^ This was completed on a standard office laptop, with processing requiring approximately an hour per data set.

GIWAXS of PCDTBT was conducted at experiment station 11–3 at the Stanford Synchrotron Radiation Lightsource at SLAC National Accelerator Laboratory. Data sets were collected using 12.7 keV X-rays with a detector distance of 316 mm and an incident angle of 0.10°. Analysis of GIWAXS data was done using Xi-cam.^34^ After an Ewald sphere curvature correction, GIWAXS images were integrated over angles −15° to 15° for in-plane scattering and 75° to 105° for out-of-plane scattering.

Samples for rheology were prepared by creating 1 mm thick discs, and experiments were set up using previously described methods.^35,36^ Bubbles were removed by heating samples under vacuum at 300 °C for 45 min, and then the samples were annealed at 190 °C for 4 h. Linear oscillatory shear measurements were performed using an ARES-G2 rheometer at a frequency of 1 rad/s with a heating/cooling rate of 5 °C/min. For low-temperature measurements, a lower molecular weight polymer was used to avoid sample delamination.

## Results

### PCDTBT Phase Behavior

Previous work disagrees on the phase behavior of PCDTBT, with reports stating that the polymer is semicrystalline^28^ or amorphous.^29^ A 2D oblique unit cell has been demonstrated, although a full 3D crystal structure has proven difficult to characterize due to a lack of long-range order in PCDTBT.^30^ Additionally, PCDTBT is widely reported to be a liquid crystalline polymer.^28–30,32^ To determine the phase behavior of PCDTBT, we use linear oscillatory shear rheology, as previously shown.^32^ Thermal transistions, including *T*_g_, are revealed in rheology data as a peak in the loss modulus corresponding to the relaxation process associated with the transition.

Figure 2a shows that at −90 °C, the storage modulus (*G′*) drops from above 1 GPa to just below that value, while the loss modulus (*G*″) has a peak with temperature. This is consistent with a *T*_g_ assocated with the side chains (*T*_g*,*s_). At 126 °C, a second *T*_g_ is observed, which corresponds to the relaxation of the backbone (Figure 2b,c). Without annealing (Figure 2b), the rheology data shows that *G*′ and *G*″ are similar until about 230 °C. The drop in *G′* and *G*″ at 230 °C may be a weak signature of crystal melting. At 276 °C, both moduli increase with temperature, which is a clear signature of a nematic-to-isotropic transition (*T*_iso_).^32^ Annealing PCDTBT at 190 °C prior to a temperature sweep leads to a *G*′ larger than *G*″ for a broad temperature range, which indicates a solid-like response (Figure 2c). Crystals melt at 231 °C (*T*_m_), as evidenced by a clear drop in the storage modulus and a peak in the loss modulus at this temperature. *G′* and *G*″ also increase at 276 °C for the sample annealed at 190 °C. We conclude that the high *T*_g_ of PCDTBT allows for kinetically trapped amorphous or liquid crystalline phases, while annealing at 190 °C allows for crystallization to occur, consistent with previous differential scanning calorimetry results.^32^

**Figure 2:**
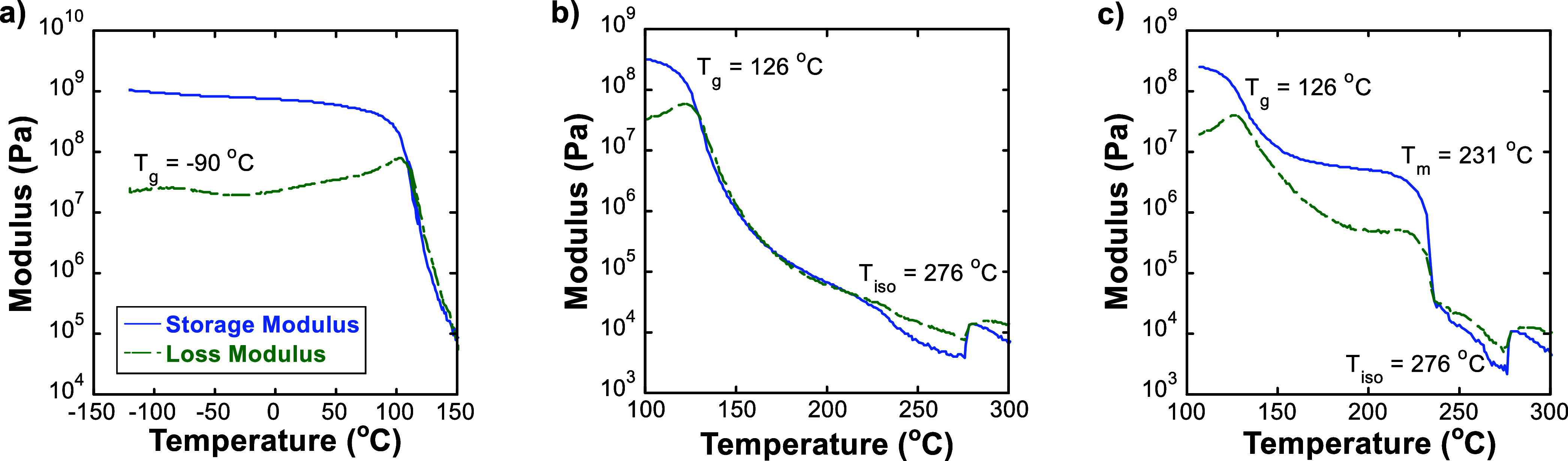
Linear oscillatory shear rheology of PCDTBT reveals thermal transitions and phase behavior. (a) Storage and loss modulus vs temperature, from ca. −120 to 150 °C (*M*_w_ = 21 kDa), and (b) from 100 to 300 °C, unannealed prior to temperature sweep. (*M*_w_ = 27 kDa). (c) Storage and loss modulus versus temperature, annealed at 190 °C prior to temperature sweep (*M*_w_ = 27 kDa).

We confirm the presence of a semicrystalline phase in PCDTBT using GIWAXS. Diffraction data from unannealed PCDTBT (Figures 3a and S1) shows two broad peaks near *q* of 0.45 Å^–1^ and 1.5 Å^–1^, corresponding to real-space dimensions of 14 Å and 4.2 Å. These spacings may represent weak correlations between adjacent chains. Annealing at 190 °C leads to an enhancement in scattering both in-plane and out-of-plane, as seen in Figure 3b. Figure 3c compares the integrated GIWAXS profiles for unannealed and annealed PCDTBT films. Annealing at 45 °C, which is below the backbone *T*_g_, leads to subtle changes in the scattering profile when compared to unannealed films. Films annealed at 190 °C show a large enhancement in the peak at *q* = 0.34 Å^–1^, corresponding to a real-space distance of 18.6 Å of backbones in the [*h*00] direction. Deconvolution of this peak estimates a lower limit of 1.6% crystallinity based on peak area. However, GIWAXS alone does not always provide an accurate quantitative measure of crystallinity.^37^

**Figure 3:**
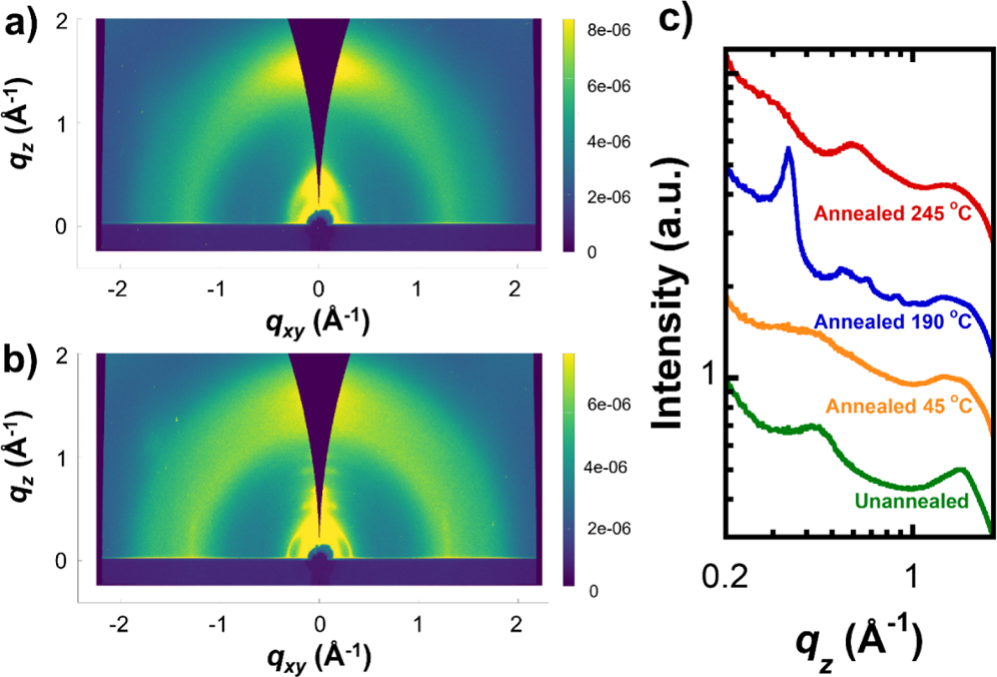
GIWAXS data shows emergence of a crystalline phase after thermal annealing. (a) GIWAXS data for unannealed PCDTBT and (b) PCDTBT annealed at 190 °C. Intensity color scale shown on right of images. (c) Out-of-plane scattering intensities versus scattering vectors for PCDTBT that is annealed under different conditions.

Various out-of-plane peaks are also apparent, which we attribute to a signature of a semicrystalline phase. A shoulder in the broad peak at 1.5 Å^–1^ near *q* ∼ 1.4 Å^–1^ corresponds to a spacing of approximately 4.4 Å. The observed values are consistent with previously reported GIWAXS data of annealed PCDTBT, which range from 0.3 to 0.35 Å^–1^ and 1.35 to 1.5 Å^–1^, respectively.^30,38^ Scherrer analysis of these peaks yields a coherence length (*L*) of 3.2 nm, or L^2^ of 10 nm^2^, which we compare to estimates of crystal size from HRTEM below.

### HRTEM Imaging of ca. 2 nm Lattice Spacings

Having established the presence of a crystalline phase in PCDTBT annealed at 190 °C, we used HRTEM to image the lattice planes. Figures 4a,b and S2 show approximately 2 nm lattice spacings within films of PCDTBT. Such a *d*-spacing in conjugated polymers is typically representative of stacking in the [*h*00] direction of crystals^30,31^ and sometimes called the “layer spacing” or “lamellar spacing”. The average full width at half max of these peaks is 1.0 nm, while simulations report bilayer backbone segments to be 0.85 nm thick.^30^ These differences could be the result of the sinusoidal curvature of the backbones blurring together during imaging, causing them to appear thicker.

**Figure 4:**
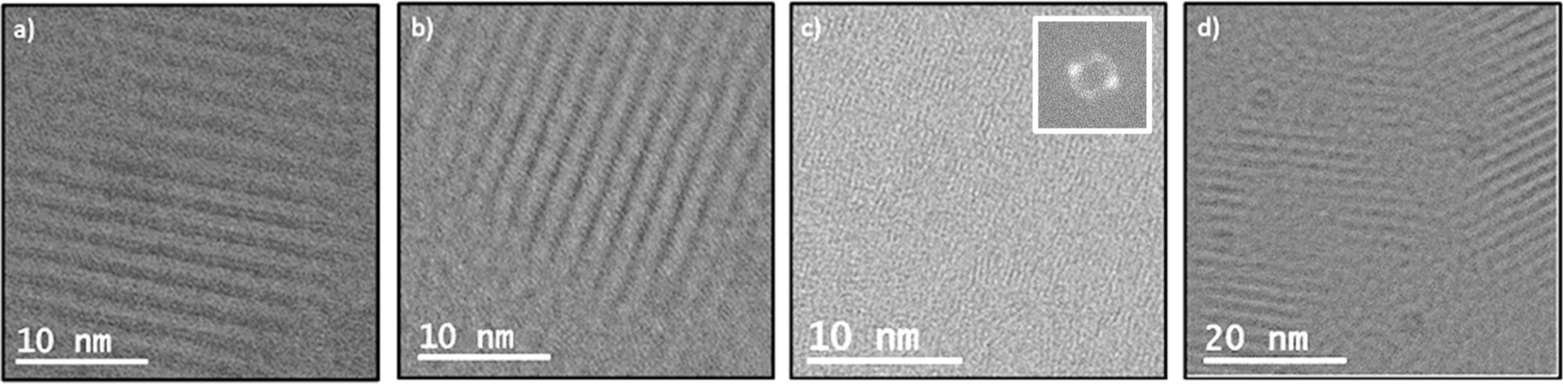
HRTEM micrographs of PCDTBT films exhibit multiple lattice spacings. Micrographs with apparent spacings near (a, b) 2 nm and (c) 0.46 nm. Fast-Fourier transform (FFT) of 0.46 nm spacings provided to aid interpretation. (d) An example of a crystal grain boundary.

Analysis of many images can help determine the frequency of each of the lattice spacings observed near 2 nm. We use our ca. 600 image TEM data set with GRATEv2 ^24,27^ to find the area of crystals with spacings near 2 nm, as shown in Figure 5. The distribution in spacings shown in Figure 5 can be described with two Gaussian peaks at average spacings of 1.98 and 2.16 nm. Because both peaks are too large to represent π–π stacking in the (010) lattice plane (typically on the order of ∼0.4 nm for conjugated polymers^2^), these peaks likely represent the lamellar spacing in the [*h*00] direction. If there are indeed two distinct spacings of 1.99 and 2.16 nm, they could represent two polymorphs of PCDTBT, as previous literature reports variations in lamellae of up to 0.4 nm depending on annealing conditions.^30^ Although the error in determining the spacing of any single grain from the apparent maximum in frequency of fast Fourier transforms is a ca. 10% for the crystal sizes examined here, Gaussian mixture modeling of the area-weighted *d*-spacing distribution strongly supports the presence of two distinct populations (AIC and BIC both favor a bimodal distribution). Further details of this calculation are provided in Materials and Methods.

**Figure 5:**
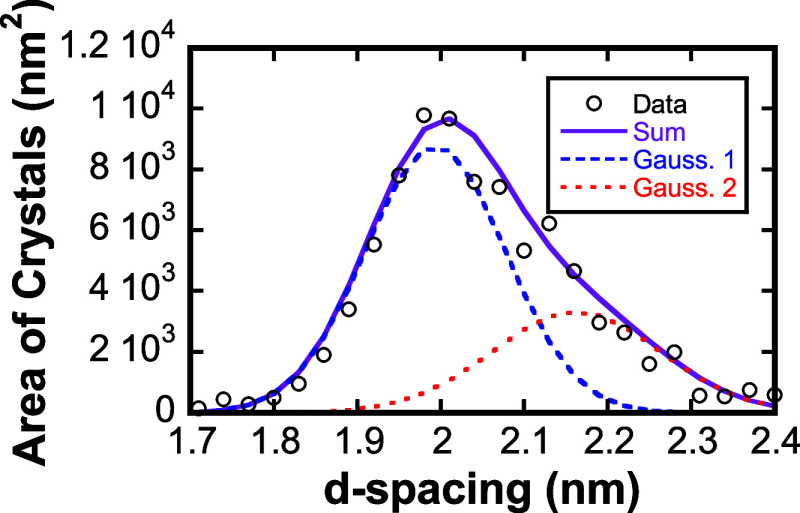
Area occupied by crystalline domains for a range of *d*-spacings near 2 nm. Data fit to two gaussians centered at 1.99 and 2.16 nm (gauss. 1, gauss. 2, respectively).

### Analysis of π–π Spacings

Through cryogenic HRTEM measurements, 0.46 nm spacings were directly imaged before samples suffered degradation due to the electron beam (Figure 4c). Across the collected images, GRATEv2 identified >400 domains in which both 0.46 and 2.0 nm lattice plains were visible in the same area. While some of these cases represent crystals stacked on top of one another in 3D space, many represent single crystals in an edge-on orientation with both their (100) and (010) lattice planes orientated along the TEM beam path. In such cases, the relative angle between the observed lattice planes, θ_r_, corresponds to the unit cell angle β between the [*h*00] and [0*k*0] directions.

For areas within our images where both 0.46 and 2.0 nm lattice plains are visible, we identify the centroid of the domain defined by each of the two lattice planes and examine whether these domains appear to overlap. We use centroids and the size of the domains to track the edge-to-edge separation distance (*r*_edge_, see Materials and Methods) such that *r*_edge_ < 0 if domains overlap but with the domain centroid not exactly on top of each other, *r*_edge_ > 0 if domains do not overlap, and *r*_edge_ = 0 if domains overlap with their centroids aligned or if the lattice spacings are from the same crystal. Figure 6 shows the joint probability distribution of θ_r_ as a function of *r*_edge_ for domains that show 0.46 and 2.0 nm lattice spacings. The probability density was weighted by *1/r*_edge_ to correct for the increasing radial area available for observation at larger separation distances (see Materials and Methods). The low probability density at *r*_edge_ < 0 suggests that this set of identified crystals contains few domains that overlap along the thickness of the film. Taking the data near *r*_edge_ = 0 as indicative of lattice spacings observable within a single crystal, we estimate the angle β between lattice planes to be 85°. This angle differs slightly from the angle β of 83.6° previously reported for the crystal structure of PCDTBT.^39^

**Figure 6:**
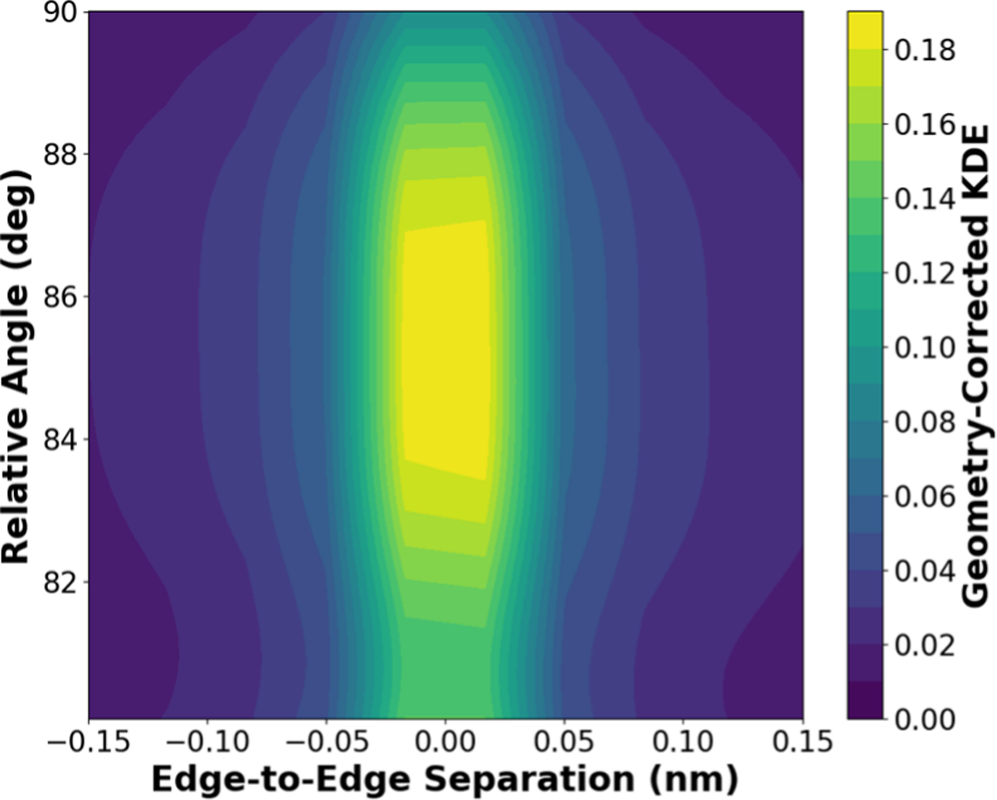
Geometry-corrected probability distribution from HRTEM images containing both (010) and (100) lattice planes within the same field of view, plotted as a function of edge-to-edge overlap distance (*r*_edge_) and relative orientation between spacings. A positive edge-to-edge overlap distance corresponds to lamellae with overlapping areas.

Based on convergent beam electron diffraction (CBED) patterns (Figure 7a) obtained during 4D-STEM scans, the π–π spacing of PCDTBT in the semicrystalline phase was measured to be 0.46 nm. This observation is confirmed by cryogenic HRTEM images, such as that in Figure 4c. This length scale is slightly larger than that seen in other conjugated polymers such as P3HT.^2^ This larger π–π stacking distance may arise from the side chains on the nitrogen atom that can rotate outside of the plane of the backbone. This additional conformational degree of freedom creates more steric hindrance, which could prevent tighter π–π stacking. This distance falls within the spectrum of previously reported X-ray scattering data for PCDTBT, ranging from 0.42 to 0.46 nm,^30,38^ and is near our value from the broad GIWAXS reflection corresponding to approximately 0.44 nm.

**Figure 7:**
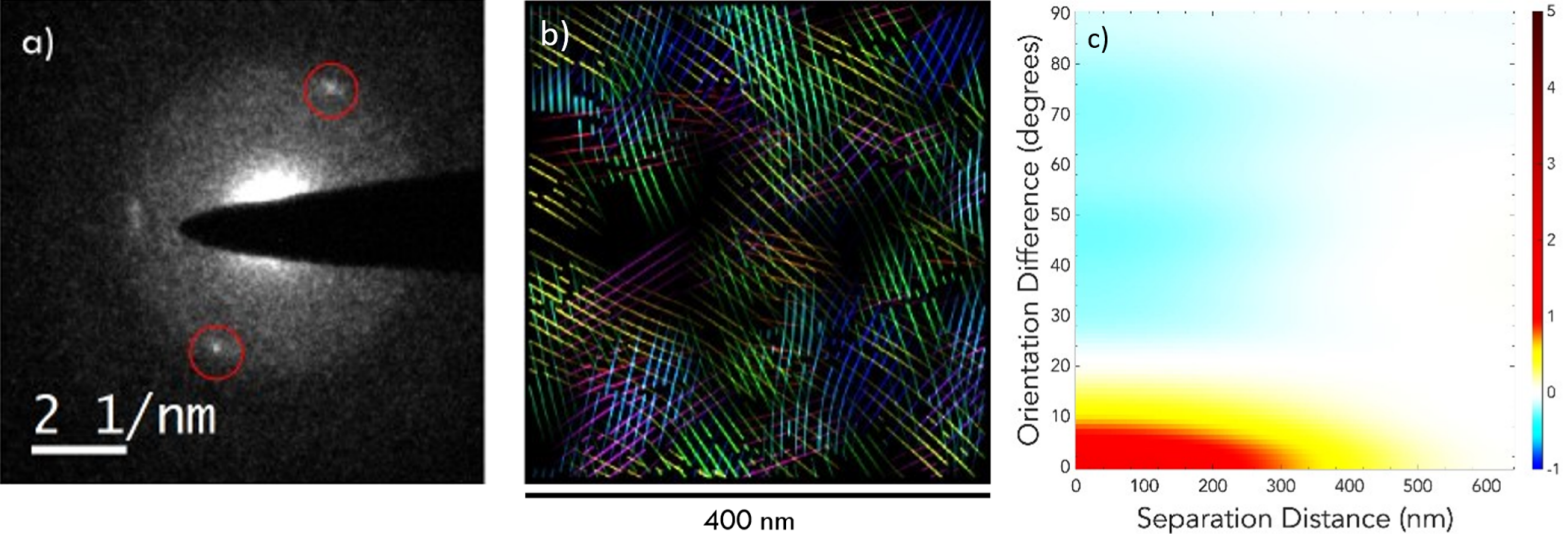
(a) CBED pattern from a PCDTBT film demonstrating the presence of diffraction spots (red circles). (b) 4D-STEM orientation map of PCDTBT, which is computationally generated from thousands of individual diffraction patterns. Lines on the orientation map do not correspond to individual polymer chains. Rather, they are a guide for the eye to demonstrate where regions with similar spacing size (0.46 nm) and orientation exist. Flow line color coding is detailed in Figure S4b. (c) A heat map demonstrating the orientation correlation strength of diffraction peaks as a function of separation distance generated from 4D-STEM data sets. The heat scale bar represents the normalized spatial autocorrelation value. Plot was generated from regions of interest measuring 1000 nm across (Figure S4a). While CBED images contain subnanometer features that enable highly precise assignment of lattice orientations (<1°), values in this plot were binned for easier interpretation.

We can map the direction of crystals by representing them as lines with different colors for different directions, as shown in Figure 7b and for a larger field of view in Figure S4a. The spacing between lines is set for visualization and does not correspond to any structural feature. We show how the orientation between individual diffraction patterns decay in Figure 7c, which shows a relatively tight distribution and loss of orientation correlations near 300 nm. These results imply either a crystal size near 300 nm, or local orientation correlations between smaller crystals, as we discuss below. The flattening of normalized spatial autocorrelation values at greater separation distances is likely due to a reduction in available samples with sufficient separation distances within a fixed scan area.

### Quantification of Morphological Features

We used our HRTEM data set and GRATEv2 to quantify additional morphologial properties of PCDTBT. Figure 8a shows the distribution of domain sizes for ordered domains with spacings of 0.46 ± 0.04 nm. This spacing was selected for quantification because of the importance of π–π stacking for intermolecular charge transport. This analysis also highlights the good agreement between GRATEv2 results and the reflections identified from 4D-STEM. Figure 8a demonstrates that the square root of domain sizes for the identified domains follows an asymmetric distribution with the primary peak centered around 3.1 nm. This corresponds to domain sizes ca. 10 nm^2^, which is in good agreement with *L* = 3.2 nm produced by Scherrer analysis of the GIWAXS data.

**Figure 8:**
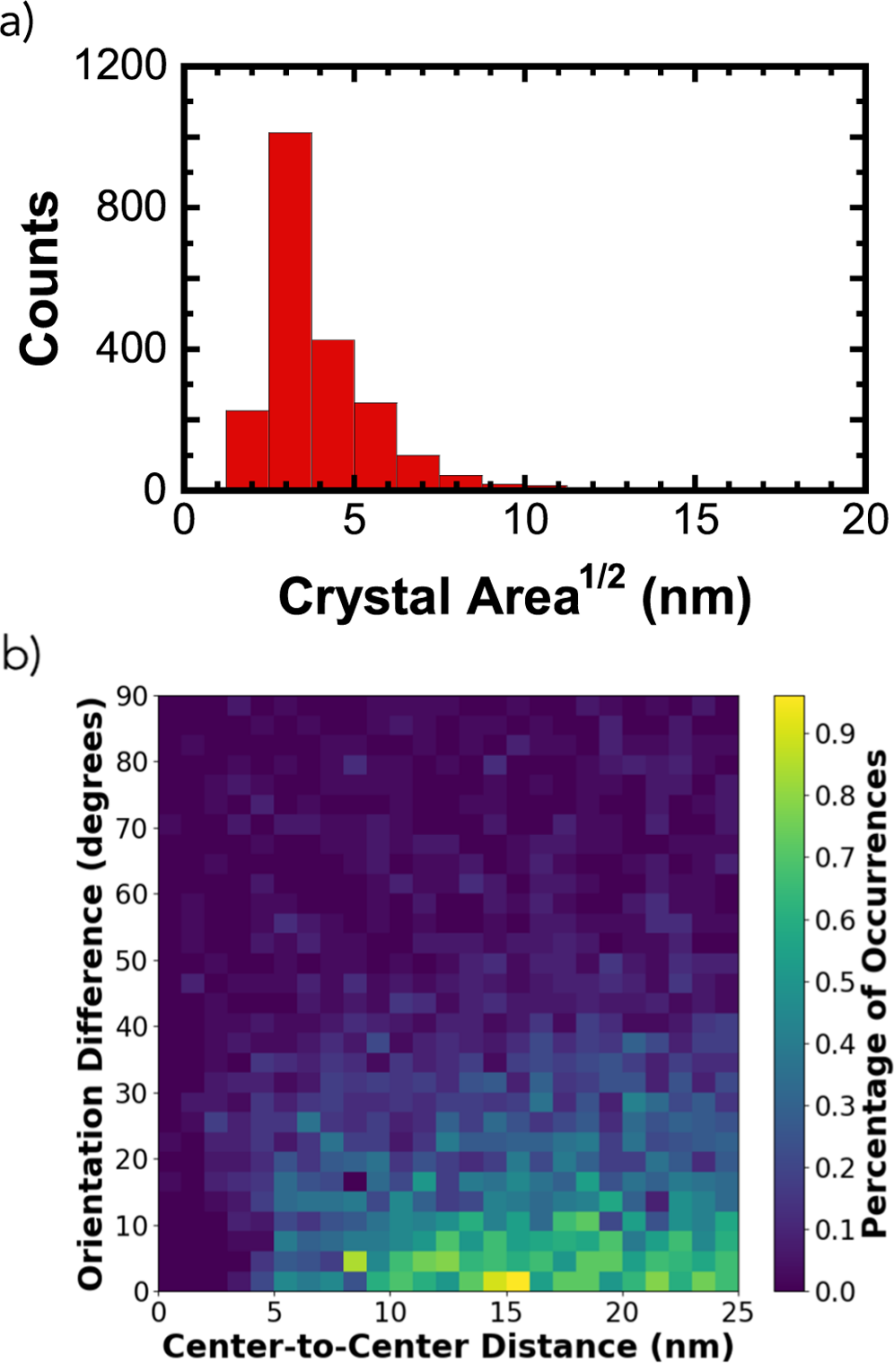
GRATEv2 analysis of HRTEM data sets showing (a) the distribution of domain sizes for all ordered domains identified as having a near 0.46 nm spacing, and (b) the relative orientation between these ordered domains as a function of separation distance. While HRTEM images contain subnanometer features that enable highly precise assignment of lattice orientations (<1°), values in this plot were binned for easier interpretation.

Given the 10 nm probe step size used in 4D-STEM, this length scale represents a fundamental resolution limit for the maps shown in Figure 7b, rendering the dimensions of these nanoscale crystals inaccessible to 4D-STEM without complementary high-resolution imaging. While it is also possible that a portion of the crystallite bends out of a zone axis that would be visible with TEM, and thus is larger than is seen in imaging, previous reports agree on a lack of long-range crystallization in PCDTBT.^30,32^

Using GRATEv2 analysis of HRTEM images, we compared the total area covered by identified ordered domains with the total area of the images collected to yield a degree of crystallinity of 10%. Crystallinity from TEM micrographs, however, is overestimated if amorphous regions lie above or below ordered domains because TEM micrographs are projection images, and it is underestimated if domains are not aligned along a zone axis that is visible in our micrographs.

Figure 8b shows the difference in lamellar orientation as a function of separation distance (center-to-center distance) based on our GRATEv2 analysis. An example of these parameters is provided in Figure S5. As grains have a mean domain size of 3.1 nm, the number of ordered domains that neighbor each other is essentially zero until the center-to-center separation distance exceeds this value. The probability of finding another grain peaks at a center-to-center spacing near 15 nm, suggesting an amorphous or poorly ordered region between crystallites. Nevertheless, we observe a correlation in orientation, where grains are nearly colinear (stronger correlations with small orientation differences in Figure 8b).

The short-range orientational correlation between domains apparent in Figure 8b can explain the long-range order that is apparent in 4D-STEM,^11^ where locally aligned domains often extend for about 300 nm (Figure 7c). Rather than large crystals that extend in the [0*k*0] direction, PCDTBT exhibits smaller crystals, mostly near 10 nm^2^, that are aligned in the same direction. We speculate that these orientation correlations are a result of liquid crystalline order that was established prior to crystallization. The presence of a nematic phase, where chain backbones are locally oriented along a single direction, may template crystals that are aligned along the same direction. Such alignment may also help with establishing tie chains between domains.

## Conclusions

Based on GRATEv2 analysis of HRTEM data sets, we establish that PCDTBT crystalline domains span only a few nanometers in the [0*k*0] direction of the lattice. These relatively small domains exhibit proximal collinearity, such that long-range order is observable using 4D-STEM. We hypothesize that the preference for the formation of a nematic phase in this material may be responsible for producing this orientation correlation, and thus long-range order. Overall, we show that automated acquisition combined with GRATEv2 and 4D-STEM enables hierarchical quantification of conjugated polymer morphology, with GRATEv2 capturing granular features at the tens-of-nanometer scale and 4D-STEM elucidating organization on the order of hundreds of nanometers.

GRATEv2 and 4D-STEM are well suited for parallel implementation. Sample preparation can be performed concurrently, as both measurements require identical specimens, and data processing for each modality requires minimal computational overhead. The primary investment lies in data acquisition, where both 4D-STEM and automated cryogenic HRTEM rely on uniquely specialized, state-of-the-art microscopes. This motivates the development of integrated instrumentation capable of both 4D-STEM acquisition and automated cryogenic HRTEM imaging, which would streamline parallel workflows.

To our knowledge, such a multi-modal approach for quantification of conjugated polymer morphology has not been previously demonstrated within the field. This approach could reveal new aspects of the microstructure of conjugated polymers, and how they affect charge transport.

## Supplementary Material





## Notes

Within the attached Supporting Information are the following: in-place GIWAXS data (Figure S1), a line profile of Figure 4a (Figure S2), demonstration of *r*_edge_ separation distance calculation for various lattices (Figure S3), long-range 4D STEM maps (Figure S4), and demonstration of parameters used for generating Figure 8 (Figure S5). Also included are parameters obtained for PCDTBT crystals from GRATEv2 analysis of HRTEM images (PDF)
(XLSX)
