# Supplementary material for: Automated TEM Reveals Intercrystalline Correlations of Conjugated Polymers
Source: Macromolecules. 2026 Feb 9;59(4):2371–8. doi: 10.1021/acs.macromol.5c02888 (PMC12947671; doi:10.1021/acs.macromol.5c02888)
Supplement: Supplementary file 1 [file ma5c02888_si_001.pdf]

Supporting Information for

Automated TEM Reveals Intercrystalline Correlations of Conjugated Polymers

Ryan A. Fair[1], Dhruv Gamdha[2], Joshua T. Del Mundo[3], Abigail M. Fenton[3], Agatha O'Connell[1], Karen C. Bustillo[4], Esther W. Gomez[3,4], Andrew M. Minor[5,6], Baskar Ganapathysubramanian[2,7], Enrique D. Gomez[1,3]*

[1]Department of Materials Science and Engineering, The Pennsylvania State University, University Park, PA 16802, USA

[2]Department of Mechanical Engineering, Iowa State University, Ames, IA 50011, USA

[3]Department of Chemical Engineering, The Pennsylvania State University, University Park, PA 16802, USA

[4]Department of Biomedical Engineering, The Pennsylvania State University, University Park, PA 16802, USA

[5]National Center for Electron Microscopy, Molecular Foundry, Lawrence Berkeley National Laboratory, Berkeley, CA 94720, USA

[6]Department of Materials Science and Engineering, University of California, Berkeley, CA 16802, USA

[7]Translational AI Research Center (TrAC), Iowa State University, Ames, IA 50011, USA

[*]Correspondence to edg12@psu.edu

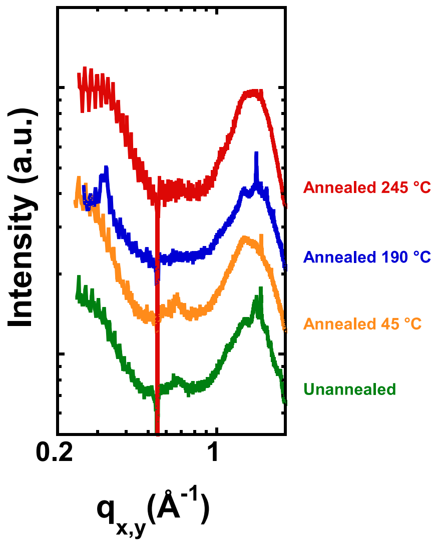

Figure S1. In-plane scattering intensities versus scattering vectors for PCDTBT that is annealed under different conditions.

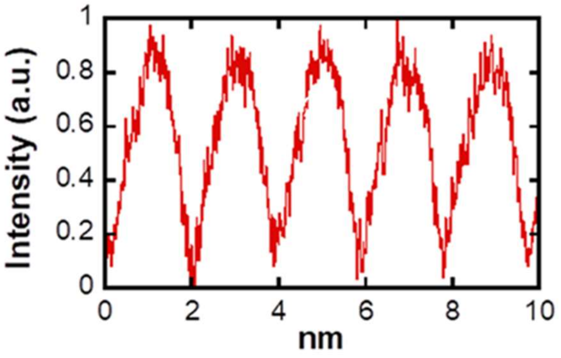

Figure S2. Line profile of image intensities in Figure 4a.

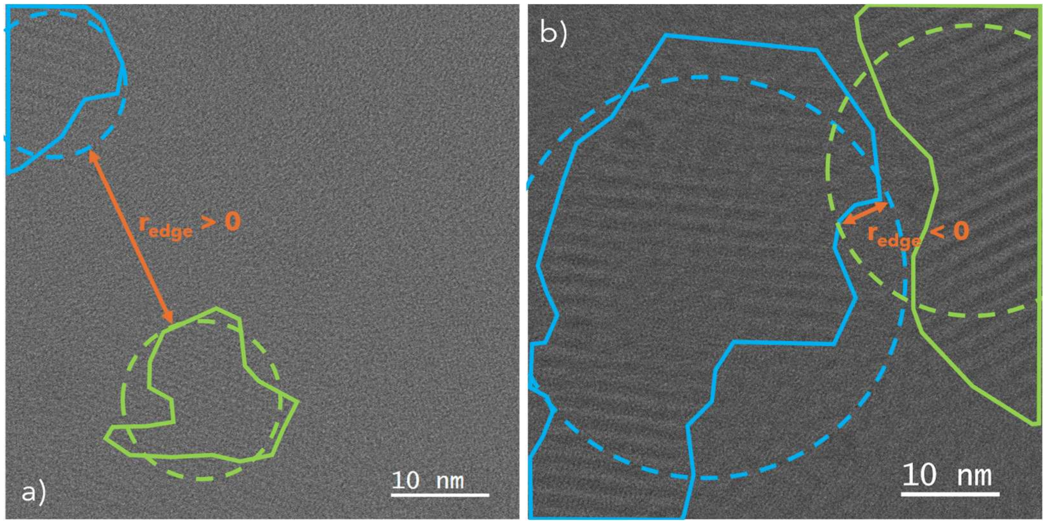

Figure S3. Demonstration of r_{edge} separation distance calculation for various lattices. Solid lines represent identified lattice area, while dotted lines represent a circular approximation of lattice geometry with radius of $r_{centroid}$. Note that $r_{edge} > 0$ for lattices with no overlap (a), and $r_{edge} < 0$ for lattices which overlap (b). Lattices in this figure were identified and labeled manually for demonstration purposes only.

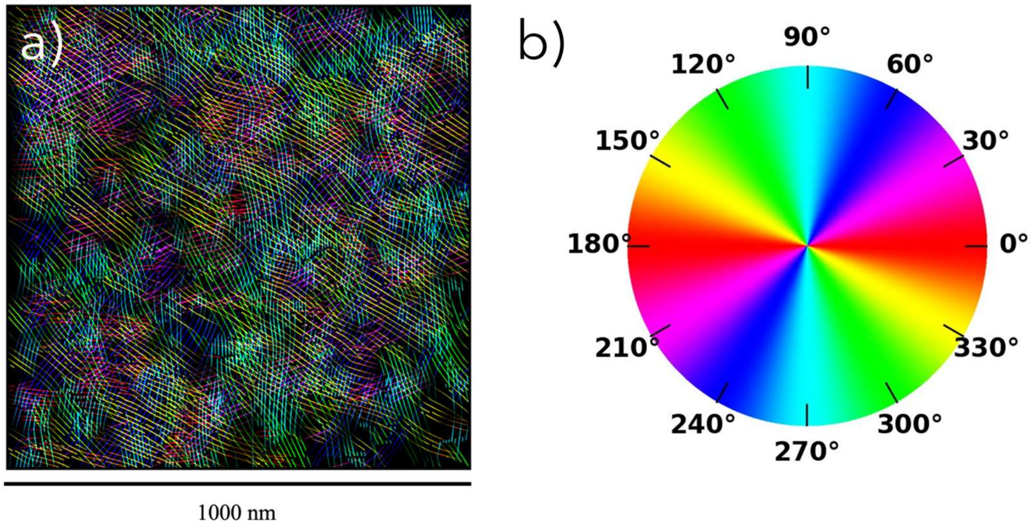

Figure S4. Long-range 4D STEM orientation map of PCDTBT annealed at 190 °C (a), and color wheel indicating flow line orientation in 4D STEM maps.

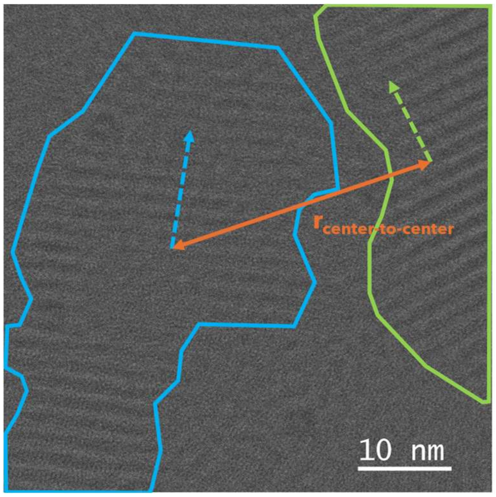

Figure S5. Demonstration of lattice orientation vectors (dotted arrows) and center-to-center distance parameter, $r_{center\text{-}to\text{-}center}$ (solid line), similar to those used for generating Figure 8. In this example, 2.0 nm lattices were used for ease of visibility. Lattices in this figure were identified and labeled manually for demonstration purposes only.
